# High‐Performance and Environmentally‐Friendly Bulk‐Wave‐Acoustofluidic Devices Driven by Lead‐Free Piezoelectric Materials

**DOI:** 10.1002/smll.202407453

**Published:** 2024-11-24

**Authors:** Wei Qiu

**Affiliations:** ^1^ Department of Biomedical Engineering Lund University Ole Römers väg 3A 223 63 Lund Sweden

**Keywords:** bulk acoustic waves, lead‐free piezoelectric materials, microscale acoustofluidics

## Abstract

Bulk‐wave‐acoustofluidic devices provide strong acoustic fields and high device efficiency, thereby offering high‐throughput capability when processing biological samples. Such devices are typically driven by lead zirconate titanate (PZT) transducers, which contain a high content of lead, inevitably resulting in environmental and biocompatibility issues. Replacing PZT with lead‐free piezoelectric materials in various ultrasonic devices is considered challenging mainly due to the inferior piezoelectric properties lead‐free materials possess compared to those of PZT. In this study, through both experiments and numerical simulations, it is demonstrated that the performance of the bulk‐wave‐acoustofluidic devices driven by (Bi,Na)TiO_3_‐BaTiO_3_‐(Bi,Na)(Mn,Nb)O_3_ (BNT‐BT‐BNMN) can match that of PZT‐driven devices at low power and is superior at intermediate power. It is found that the low acoustic impedance and the weak transverse mode in BNT‐BT‐BNMN compensate for the inferior piezoelectric properties at low power. The fact that the BNT‐BT‐BNMN devices outperform at intermediate power is consistent with the superior performance of the Mn‐doped BNT‐based piezoelectric materials compared to PZT at high power. Perfect focusing on 5‐μm‐diameter polystyrene particles at a flow rate of up to 10 mL min^−1^ is achieved using the BNT‐BT‐BNMN device at input power of 1 W.

## Graphical abstract

It is demonstrated that bulk‐wave‐acoustofluidic devices driven by (Bi,Na)TiO_3_‐BaTiO_3_‐(Bi,Na)(Mn,Nb)O_3_ (BNT‐BT‐BNMN) perform equally well as lead zirconate titanate (PZT)‐driven devices at low power and outperform them at intermediate power. The BNT‐BT‐BNMN device achieves perfect focusing of 5‐µm‐diameter polystyrene particles at a flow rate of 10 mL min^−1^ with input power of 1 W.

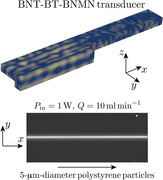

## 1 Introduction

Microscale acoustofluidics, an increasingly important area within microfluidics, uses ultrasound to manipulate particles and fluids in micro‐ and nanoscale. It has made significant progress since its emergence in early 2000. Its ability to separate,^[^ ^1^, ^2^, ^3^, ^4^, ^5^ ^]^ pattern,^[^ ^6^, ^7^, ^8^ ^]^ tweeze,^[^ ^9^, ^10^, ^11^ ^]^ trap,^[^ ^12^, ^13^ ^]^ concentrate,^[^ ^14^, ^15^, ^16^, ^17^ ^]^ and wash^[^ ^18^ ^]^ biological samples in a gentle and label‐free manner clearly highlights its potential for applications in life sciences. These functions are achieved via two physical phenomena, i.e. acoustic radiation force originating from the wave‐particle scattering and acoustic streaming arisen from the dissipation of acoustic energy in viscous fluids, when a sound field is excited in a microchannel.

The sound fields in acoustofluidic devices are typically excited by either surface acoustic waves (SAW) or bulk acoustic waves (BAW). While SAW devices offer excellent flexibility in tailoring the sound fields for various manipulation tasks,^[^ ^19^, ^20^ ^]^ they are limited by low acoustic energy density in the channel and poor device efficiency. This is primarily due to the close acoustic impedance between the medium and the polydimethylsiloxane (PDMS) material surrounding the channel, which prevents the storage of acoustic energy in the fluid. Although efforts have been made to address this issue, such as by inserting a glass lid^[^ ^21^ ^]^ or replacing PDMS with stiffer materials,^[^ ^22^ ^]^ SAW devices have not yet achieved the high throughput and efficiency required to meet clinical standards. In contrast, BAW devices exhibit much higher acoustic energy density, owing to the mismatch in acoustic impedance between the medium and surrounding materials (typically silicon or glass), leading to strong acoustic radiation forces exerting on particles and hence high throughput. Recent advances in optimizing actuation schemes have further enhanced device efficiency, enabling flow rates on the order of milliliters per minute when handling microparticles.^[^ ^23^ ^]^

Unlike lithium niobate, which is used in SAW devices, BAW devices are driven by lead zirconate titanate (PZT) transducers that contain about 60% lead by mass. The use of lead in these devices inevitably raises environmental concerns, as it does not comply with the Restriction of Hazardous Substances (RoHS) Directive established by the EU in 2003,^[^ ^24^ ^]^ as well as legislation from other countries. However, replacing PZT with lead‐free piezoelectric materials in various ultrasonic devices, particularly in actuation devices, has proven extremely challenging. This is mainly due to the inferior piezoelectric properties of lead‐free materials compared to PZT. Numerous attempts have been made to develop lead‐free alternatives for Langevin transducers,^[^ ^25^, ^26^ ^]^ ultrasonic motors,^[^ ^27^, ^28^, ^29^, ^30^ ^]^ and high‐intensity focused ultrasound transducers^[^ ^31^, ^32^ ^]^ operating at the frequencies ranging from kilohertz to megahertz, but the majority of the devices driven by lead‐free materials still cannot deliver performance comparable to PZT‐driven devices.

In this work, we compare the performance of BAW acoustophoresis devices driven by PZT and by (Bi,Na)TiO_3_‐BaTiO_3_‐(Bi,Na)(Mn,Nb)O_3_ (BNT‐BT‐BNMN) through both experiments and numerical simulations. We find that the devices driven by BNT‐BT‐BNMN perform equally well as the PZT‐driven devices at low power, as evidenced by the similar acoustic energy densities generated under the same input power, resulting in comparable device efficiencies. A clear difference in focusability is observed between PZT and BNT‐BT‐BNMN devices at input power of 1 W and high flow rates, when focusing 5‐μm‐diameter polystyrene particles. The results indicate that the performance of BNT‐BT‐BNMN devices is superior to that of PZT devices under intermediate power. The factors contributing to the high performance of the BNT‐BT‐BNMN devices at low and intermediate power are analyzed in detail.

## 2 Experimental Section

### 2.1 Selection of Piezoelectric Materials

Bulk acoustic wave (BAW) devices typically operate at resonance, where the resonance mode in the channel is excited by a specific, complex vibration mode of the solid materials. As a result, hard‐type piezoelectric materials are required for BAW devices, as they possess high mechanical *Q*‐factor *Q*_m_ and low dielectric loss factor tanδ, though their piezoelectric charge constants are lower than those of soft‐type piezoelectric materials. Those selection criteria for piezoelectric materials also apply to power ultrasonic devices and ultrasonic actuators operating under resonance conditions. For this comparison, Pz26 (Ferroperm Piezoceramics, Kvistgaard, Denmark), a typical type of hard PZT widely used in BAW devices, was selected.

The selection of hard‐type lead‐free piezoelectric materials is more challenging, as the majority of lead‐free materials developed in both academia and industry are soft‐type. One option is LiNbO_3_, which is typically used to excite surface acoustic waves. However, it suffers from the low piezoelectric charge constant *d*_33_, so it is usually not operated at its thickness mode. Since the resonance frequency of the transducer's thickness mode is often used to match the resonance frequency in the channel when designing BAW devices,^[^ ^33^ ^]^ *d*_33_ is a critical material property that should not be too low. The other two common types of lead‐free piezoelectric materials are (K,Na)NbO_3_‐based (KNN) and (Bi,Na)TiO_3_‐based (BNT) systems, with the BNT‐based system being more extensively studied for hard piezoelectric materials than the KNN‐based system. It has been found that Mn‐doping in BNT‐based system can significantly enhance the *Q*_m_ and reduce the tan*δ*.^[^ ^34^, ^35^ ^]^ Moreover, a higher *Q*_m_ than that of hard PZT can be achieved under high‐power conditions, while the heat dissipation and resonance frequency shifts are remarkably suppressed.^[^ ^36^, ^37^ ^]^ These features indicate that Mn‐doped BNT‐based piezoelectric materials are ideal for BAW devices. In this study, (Bi,Na)TiO_3_‐BaTiO_3_‐(Bi,Na)(Mn,Nb)O_3_ (BNT‐BT‐BNMN)^[^ ^36^ ^]^ developed and commercialized by Honda Electronics (HC‐70BN, Honda Electronics, Toyohashi, Japan), which also possesses good biocompatibility,^[^ ^38^, ^39^, ^40^ ^]^ was chosen. Key material properties of Pz26 and HC‐70BN are summarized in **Table** **1**. It should be noted that the material properties provided by the manufacturers are measured at low power levels and, therefore cannot accurately reflect the properties at intermediate or high power levels due to the nonlinearity of piezoelectric materials. Despite the fact that most of the piezoelectric properties of BNT‐BT‐BNMN under low‐power conditions are inferior to those of hard PZT, it remains the best hard lead‐free piezoelectric material available on the market.

**Table 1:** Material properties of PZT (Pz26) and BNT‐BT‐BNMN (HC‐70BN) obtained from Ferroperm Piezoceramics and Honda Electronics.

		PZT	BNT‐BT‐BNMN
Relative permittivity	$\varepsilon_{33}^{T}/\varepsilon_{0}$	1330	520
	$\varepsilon_{11}^{T}/\varepsilon_{0}$	1190	600
Electromechanical coupling coefficient	*k*_31_	0.327	0.09
	*k*_33_	0.684	0.4
	*k*_15_	0.553	0.37
Frequency constant (Hz·m)	*N*_31_	1500	2340
	*N*_33_	1800	2250
	*N*_15_	1020	1420
Piezoelectric charge constant (× 10^−12^ m V^−1^)	*d*_31_	−128	−30
	*d*_33_	328	110
	*d*_15_	327	153
Elastic compliance under constant electric field (× 10^−12^ m^2^ N^−1^)	$s_{11}^{E}$	13.0	8.1
	$s_{12}^{E}$	−4.35	−2.1
	$s_{13}^{E}$	−7.05	−1.5
	$s_{33}^{E}$	19.6	8.9
	$s_{44}^{E}=s_{55}^{E}$	33.2	22.7
	$s_{66}^{E}$	34.7	20.3
Density (kg m^−3^)	ρ	7700	5500
Mechanical *Q*‐factor	*Q*_m_	3300	500
Dielectric loss factor	tan*δ*	0.003	0.006
Curie temperature (°C)	*T*_C_	330	260

### 2.2 Materials and Setup

**Figure 1:**
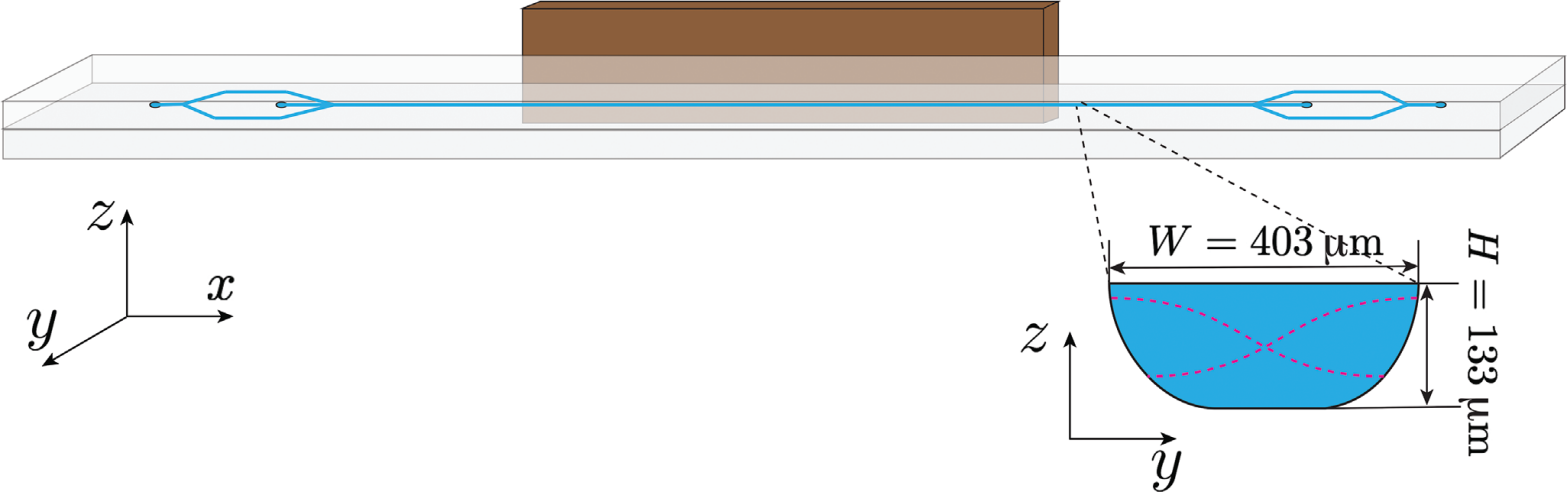
Sketch of the acoustophoresis glass chip (light gray) with dimensions of 70 × 3 × 1.1 mm^3^. The long straight channel (light blue) was isotropically etched into the bottom glass layer, with dimensions of 45 × 0.403 (top width) × 0.133 mm^3^. The piezoelectric transducer (brown) was attached to the sidewall. The dimensions of PZT and BNT‐BT‐BNMN transducers were 25 × 2 × 1 mm^3^ and 25 × 2 × 1.2 mm^3^, respectively, both corresponding to a fundamental thickness mode around 2 MHz. A half‐wavelength standing‐wave field is indicated by the dashed magenta lines in the channel cross‐section, as shown in the inset. Three chips were tested using the same transducer material.

All experiments were performed using BAW devices made of glass with outer dimensions of 70 × 3 × 1.1 mm^3^, as shown in **Figure** **1**. The microchannel was isotropically etched with the resulting dimensions of length *L* = 45 mm, top width W=403μm, and height H=133μm. Trifurcations were fabricated at the ends of the long straight channel, which were connected to two inlets and outlets. Silicone tubing (outer diameter 3 mm, inner diameter 1 mm, length 7 mm) was attached to the inlets and outlets using silicone glue (ELASTOSIL A07 TRANSLUCENT, Wacker Chemie, Munich, Germany). Six devices with identical chip and channel dimensions were prepared, three of which were driven by PZT while the other three by BNT‐BT‐BNMN.

A small piezoelectric transducer (length *L*_piezo_ = 25 mm, width *W*_piezo_ = 2 mm) was glued to one of device sidewalls using cyanoacrylate glue (Loctite Super Glue, Henkel Norden AB, Stockholm, Sweden), as illustrated in Figure 1. Side actuation in such devices has been shown to be an efficient approach, leading to high acoustic energy density in the channel and high device efficiency.^[^ ^23^ ^]^ Since PZT and BNT‐BT‐BNMN have different longitudinal and transverse speed of sound, the thickness of the transducers required to excite the fundamental thickness mode at around 2 MHz also differs (1 mm for PZT transducers and 1.2 mm for BNT‐BT‐BNMN transducers). The device admittance spectrum was measured using an impedance analyzer (IM7581, Hioki E.E. Corporation, Nagano, Japan) while the channel was filled with water. The piezoelectric transducer was driven by a function generator (33220, Agilent Technologies, Inc., Santa Clara, California) during the measurements of acoustic energy density in the channel. A power amplifier (75A250A, Amplifier Research, Souderton, Pennsylvania) was used for the particle‐focusing experiments at high flow rates. The waveforms of the applied voltage across the transducer and the resulting current were measured using voltage and current probes and displayed via a PicoScope (5244D, Pico Technology, Cambridgeshire, United Kingdom), from which the input power to the piezoelectric transducer was calculated. Fluorescent polystyrene particles with a nominal diameter of 5 μm (G0500B, Fluoro–Max, Thermo Fisher Scientific, Waltham, Massachusetts) were used for both measuring the acoustic energy density and performing the in‐flow particle focusing experiments. The particles were diluted in Milli‐Q water to achieve a suspension volume fraction of 0.01%, which is well below the threshold for inducing particle‐particle interaction.^[^ ^41^ ^]^

A spinning disk (X‐Light V3, Crest, Rome, Italy) confocal microscope (Eclipse Ti2, Nikon, Tokyo, Japan) equipped with a CMOS camera (Prime 95B, Teledyne Photometrics, Tucson, Arizona) was used for all the experiments. A 10× objective lens with a numerical aperture of 0.3 was employed, providing a field of view of 1.83 × 1.83 mm^2^ and an optical slice thickness of 5.7 μm. Fluorescent particles were excited by a laser diode (CELESTA Light Engine, Lumencor, Beaverton, Oregon) with a peak wavelength of 488 nm. The excitation light passed through an excitation filter with a passband from 475 to 490 nm, while the emission light from the fluorescent particles passed through an emission filter with a passband from 510 to 531 nm. A photograph of the experimental setup is shown in Figure S1 (Supporting Information).

### 2.3 Experimental Approaches for Comparing Device Performance

To compare the performance of the devices, the optimal frequency of each device was first determined through the in‐flow particle focusing experiment. For BAW devices, the resonance frequency can be used to drive the device, and it can be found by locating the peak of the total acoustic energy within a frequency range under a constant applied voltage. Alternatively, a frequency at which the device exhibits the highest efficiency can also be used as the operating frequency. This can be identified by locating the peak of the total acoustic energy within a frequency range under a constant input power. Since acoustofluidic devices are often designed for biomedical applications where biological samples are handled, it is important to avoid significant temperature rise, making efficient driving necessary. In this study, the frequency at which the device efficiency is the highest was chosen as the operating frequency. This frequency can be determined by flowing 5‐μm‐diameter polystyrene particles through the channel at a constant flow rate and with constant power supplied to the transducer at different frequencies. The particles were introduced into the channel using a syringe pump (neMESYS, Cetoni GmbH, Korbussen, Germany). The particle focusing bandwidth was characterized by plotting the intensity profile near the center outlet, and the full width at half maximum (FWHM) was calculated. A smaller FWHM indicates higher total acoustic energy in the channel, and hence, higher device efficiency. For each device, this investigation was conducted over a frequency range from 1.8 to 2.1 MHz.

Once the frequency corresponding to the highest device efficiency was identified, the acoustic energy density *E*_ac_ along the entire actuation zone (25 mm long) in each device under 12.5 mW input power was measured. This was determined by measuring the focusing velocity of 5‐μm‐diameter polystyrene particles under stop‐flow conditions using confocal micro‐particle image velocimetry (μPIV).^[^ ^23^ ^]^ The principle of this measurement has been described in previous studies^[^ ^23^, ^42^, ^43^ ^]^ and is briefly outlined here. A particle suspended in a sound field experiences an acoustic radiation force $F_{\mathrm{rad}}$ due to the wave‐particle scattering. For a half‐wavelength standing‐wave field along the channel width (*y*) direction $e_{y}$, the analytical solution for $F_{\mathrm{rad}}$ on a compressible spherical particle of radius *a* was derived by Yosioka and Kawasima,^[^ ^44^ ^]^ as well as by Gor'kov,^[^ ^45^ ^]^ in an inviscid fluid. The expression for $F_{\mathrm{rad}}$, accounting for medium viscosity, is given by ref. [46]

(1) $$\begin{array}{cc} F_{\mathrm{rad}}\left(y\right) & =4\pi a^{3}k_{y}E_{\mathrm{ac}}\Phi \left(\tilde{\kappa },\tilde{\rho },\tilde{\delta }\right)\sin\left(2k_{y}y\right)e_{y} \end{array}$$

(2) Φκ∼,ρ∼,δ∼=13f1κ∼+12Ref2ρ∼,δ∼

where *k*_y_ and Φ are the wavenumber and acoustic contrast factor, respectively. The monopole and dipole scattering coefficients *f*_1_ and *f*_2_ are given by

(3) $$\begin{array}{cc} f_{1}\left(\tilde{\kappa }\right)=1-\tilde{\kappa },\;f_{2}\left(\tilde{\rho },\tilde{\delta }\right) & =\frac{2\left[1-\Gamma \left(\tilde{\delta }\right)\right]\left(\tilde{\rho }-1\right)}{2\tilde{\rho }+1-3\Gamma \left(\tilde{\delta }\right)} \end{array}$$

where $\Gamma \left(\tilde{\delta }\right)=-\frac{3}{2}\left[1+i\left(1+\tilde{\delta }\right)\right]\tilde{\delta }$, with $\tilde{\kappa }=\frac{\kappa_{p}}{\kappa_{0}}$, $\tilde{\rho }=\frac{\rho_{p}}{\rho_{0}}$, and $\tilde{\delta }=\frac{\delta }{a}$ ($\delta =\sqrt{2\eta_{0}/\left(\rho_{0}\omega \right)}$ is the thickness of the viscous boundary layer). Here, ρ_p_, ρ_0_, κ_p_, κ_0_, and η_0_ are the density of the particle and the medium, the compressibility of the particle and the medium, and the medium dynamic viscosity, respectively. When the particle is set in motion, $F_{\mathrm{rad}}$ balances the Stokes drag force $F_{\mathrm{drag}}$, hence the particle quickly reaches its terminal velocity v and its acceleration can be neglected. For a spherical particle moving with a velocity v in a quiescent fluid, $F_{\mathrm{drag}}$ is expressed as

(4) $$F_{\mathrm{drag}}=6\pi \eta_{0}av$$

By balancing Equations (1) and (4), v in *y*‐direction $v_{y}$ can be obtained as

(5) $$v_{y}=\frac{2a^{2}k_{y}E_{\mathrm{ac}}\Phi \left(\tilde{\kappa },\tilde{\rho },\tilde{\delta }\right)\sin\left(2k_{y}y\right)e_{y}}{3\eta_{0}}$$

Thus, *E*_ac_ can be calculated by measuring $v_{y}$ using μPIV. It should be noted that the contribution of acoustic streaming to $v_{y}$ is neglected, as it is sufficiently small for polystyrene particles of this size in water at 2 MHz.^[^ ^47^, ^48^, ^49^ ^]^ As detailed in ref. [23], using confocal μPIV offers the advantage of excluding particles moving near the channel ceiling and bottom, whose motion is influenced by particle‐wall interactions.^[^ ^50^, ^51^, ^52^, ^53^ ^]^ The μPIV experiments were conducted for all six devices over a 25‐mm‐long region where the transducer was mounted, requiring measurements in 14 separate sections based on the field of view provided by our microscope and camera. In each section, particle focusing was repeated 11 to 37 times to improve the accuracy. PIVlab, an open‐source PIV algorithm based on MATLAB, was used to process the data.^[^ ^54^ ^]^

## 3 Numerical Model

The numerical simulations were performed using the finite element software COMSOL Multiphysics 6.0,^[^ ^55^ ^]^ in three dimensions (3D), and the details are presented in this section.

**Figure 2:**
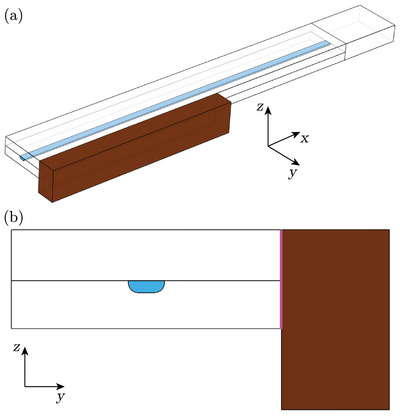
a) Sketch of the 3D model where a long, straight microchannel (light blue) was embedded in the lower glass layer. The piezoelectric transducer (brown) was mounted on the side of the device via a glue layer with an offset. Only half of the device was modeled, with symmetric boundary conditions applied to the *yz*‐plane. b) Cross‐sectional view of the 3D model on the *yz*‐plane, with the glue layer visualized in magenta.

The 3D model of the acoustofluidic device included a long, straight channel embedded in the glass, as shown in **Figure** **2**a. A piezoelectric transducer was mounted on one of the device's sidewalls with an offset along the height (*z*) direction. The dimensions of the channel, device, and transducer were the same as those used in experiments, except that the trifurcations at the channel ends were not modeled. A thin (5‐μm‐thick) glue layer was placed between the device and the transducer, as depicted in Figure 2b. The material properties of water, glass, and the glue layer are summarized in **Table** **2**, while the properties of the piezoelectric transducers can be found in Table 1. In this simulation, the strain‐charge form of the piezoelectric equations was used to calculate the electromechanical coupling of the piezoelectric transducer, expressed in Voigt notation as

(6) $$\left(\begin{array}{c} S_{\mathrm{xx}}\\ S_{\mathrm{yy}}\\ S_{\mathrm{zz}}\\ S_{\mathrm{yz}}\\ S_{\mathrm{xz}}\\ S_{\mathrm{xy}} \end{array}\right)=\left(\begin{array}{cccccc} s_{11} & s_{12} & s_{13} & 0 & 0 & 0\\ s_{12} & s_{11} & s_{13} & 0 & 0 & 0\\ s_{13} & s_{13} & s_{33} & 0 & 0 & 0\\ 0 & 0 & 0 & s_{44} & 0 & 0\\ 0 & 0 & 0 & 0 & s_{44} & 0\\ 0 & 0 & 0 & 0 & 0 & s_{66} \end{array}\right)\left(\begin{array}{c} T_{\mathrm{xx}}\\ T_{\mathrm{yy}}\\ T_{\mathrm{zz}}\\ T_{\mathrm{yz}}\\ T_{\mathrm{xz}}\\ T_{\mathrm{xy}} \end{array}\right)+\left(\begin{array}{ccc} 0 & 0 & d_{31}\\ 0 & 0 & d_{31}\\ 0 & 0 & d_{33}\\ 0 & d_{15} & 0\\ d_{15} & 0 & 0\\ 0 & 0 & 0 \end{array}\right)\left(\begin{array}{c} E_{x}\\ E_{y}\\ E_{z} \end{array}\right)$$

(7) $$\left(\begin{array}{c} D_{x}\\ D_{y}\\ D_{z} \end{array}\right)=\left(\begin{array}{cccccc} 0 & 0 & 0 & 0 & d_{15} & 0\\ 0 & 0 & 0 & d_{15} & 0 & 0\\ d_{31} & d_{31} & d_{33} & 0 & 0 & 0 \end{array}\right)\left(\begin{array}{c} T_{\mathrm{xx}}\\ T_{\mathrm{yy}}\\ T_{\mathrm{zz}}\\ T_{\mathrm{yz}}\\ T_{\mathrm{xz}}\\ T_{\mathrm{xy}} \end{array}\right)+\left(\begin{array}{ccc} \varepsilon_{11}^{T} & 0 & 0\\ 0 & \varepsilon_{11}^{T} & 0\\ 0 & 0 & \varepsilon_{33}^{T} \end{array}\right)\left(\begin{array}{c} E_{x}\\ E_{y}\\ E_{z} \end{array}\right)$$

Here, the strain, stress, electric field, and electric displacement field are denoted as S, T, E, and D, respectively.

**Table 2:** Material properties used in the model provided at room temperature.

Water		
Density	998	kg m^−3^
Speed of sound	1497	m s^−1^
Dynamic viscosity	0.893	mPa s
Bulk viscosity	2.85	mPa s
Pyrex glass		
Density	2240	kg m^−3^
Poisson's ratio	0.245	
Young's modulus	60(1+i/2420)	GPa
Glue layer		
Thickness	5	μm
First Lamé parameter	4.82(1+i/10)	GPa
Second Lamé parameter	2.06(1+i/10)	GPa

The stress and displacement fields across the solid–solid (glass‐transducer) and solid‐fluid (glass‐water) interfaces were continuous. Zero stress was applied on the outer boundaries facing the air. An alternating electric potential with an amplitude of 2.5 V_pp_ was applied to the piezoelectric transducer. Although acoustic streaming was not calculated in this simulation, damping in the viscous boundary layers was included, as it is one of the major damping mechanisms in acoustofluidic devices.^[^ ^56^ ^]^ However, resolving the thin boundary layers with fine mesh elements significantly increases the computational cost, particularly in 3D models, which is impractical for our case. Therefore, we applied an effective boundary condition in the fluid domain that solves the boundary‐layer fields analytically.^[^ ^57^ ^]^ This boundary condition has been implemented in COMSOL Multiphysics 6.0. Symmetric boundary conditions were applied on the *yz*‐plane, so only half of the device was modeled. In addition to the losses in viscous boundary layers, the other damping mechanisms in the model included the internal friction losses in the glass, the dielectric and vibration losses in the piezoelectric transducer, and losses in the glue layer. Viscous damping in the bulk of the fluid, thermoviscous effects, and air damping were not considered in the model.

The simulation was performed on a Hephaestus personal computer with 256 GB of RAM and an AMD Ryzen Threadripper 3970X 32‐Core Processor (clock frequency of 3.69 GHz). Triangular mesh elements were generated with a maximum element size of $h_{\mathrm{fl}}=26\;\mu m$ in the fluid domain, $h_{\mathrm{glass}}=180\;\mu m$ in the glass, $h_{\mathrm{PZT}}=120\;\mu m$ in the PZT transducer, and $h_{\mathrm{BNT}}=140\;\mu m$ in the BNT‐BT‐BNMN transducer. These mesh element sizes were finer compared to those used in our previous 3D model.^[^ ^58^ ^]^ A standard mesh convergence test was performed following the approach described by Muller et al.^[^ ^48^ ^]^ to confirm that the mesh element sizes were appropriate. Details regarding the mesh convergence test can be found in Section S2 (Supporting Information). The mesh configurations resulted in a total of 2.3 × 10^6^ mesh elements and 7.3 × 10^6^ degrees of freedom for the model with the PZT transducer, and a total of 2.2 × 10^6^ mesh elements and 6.9 × 10^6^ degrees of freedom for the model with the BNT‐BT‐BNMN transducer. The computation time for each frequency was about 47 min for the model with the PZT transducer and about 35 min for the model with the BNT‐BT‐BNMN transducer.

## 4 Results and Discussion

**Figure 3:**
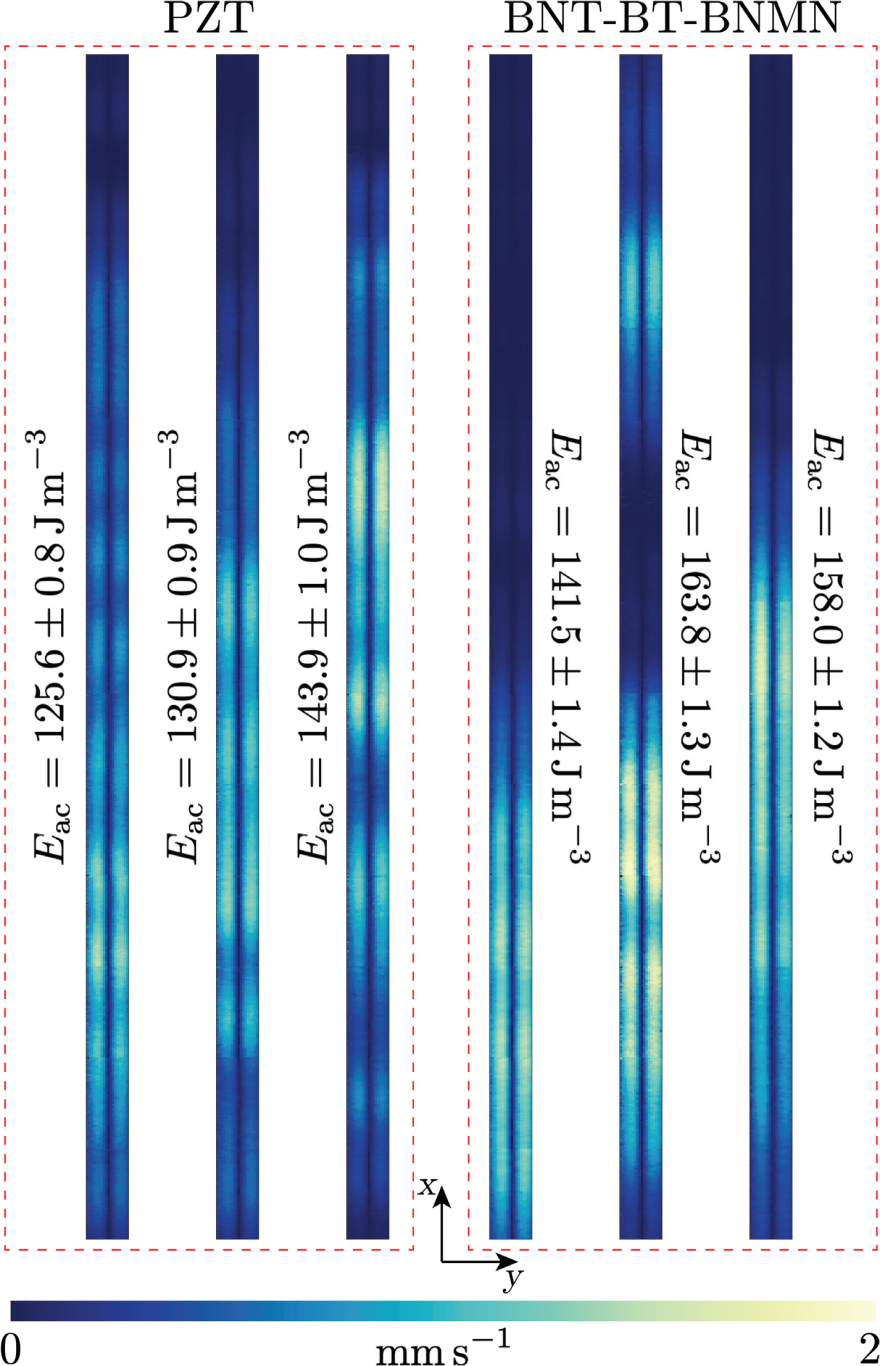
Measurements of *E*_ac_ using μPIV in three devices driven by PZT and three devices driven by BNT‐BT‐BNMN. The measurements were conducted in a 25‐mm‐long region of the channel where the transducer was located, under *P*_in_=12.5 mW. The amplitude of the particle‐focusing velocity along the channel width (*y*) direction is indicated by the color plot, ranging from 0 (dark blue) to 2.04 mm s^−1^ (yellow). The *y*‐dimension is expanded by a factor of three for better visibility.

**Figure 4:**
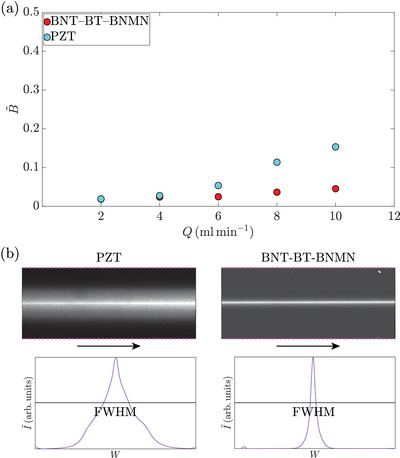
a) Measured $\tilde{B}$ [full width at half maximum (FWHM) normalized by the channel width at mid‐height] versus flow rate *Q*. The measurements were performed with one PZT device and one BNT‐BT‐BNMN device under *P*_in_ = 1 W. b) The upper row shows the particle focusing band near the center outlet at a flow rate of 10 mL min^‒1^, averaged over 50 frames. The arrows indicate the flow direction, and the channel sidewalls are marked by magenta dashed lines. The lower row shows the corresponding intensity profile of the particle focusing band, from which the FWHM was calculated.

The acoustic energy density *E*_ac_, measured at input power *P*_in_ = 12.5 mW along the 25‐mm‐long actuation zone of all six devices, is illustrated in **Figure** **3**. Surprisingly, the *E*_ac_ produced by the BNT‐BT‐BNMN devices was comparable to that of the PZT devices, despite the inferier piezoelectric properties of BNT‐BT‐BNMN compared to PZT. This suggests that the device efficiencies were similar, as the operating frequency used in this comparison corresponds to the frequency at which each device achieved its highest efficiency. As described in several previous works,^[^ ^23^, ^42^, ^59^ ^]^ achieving a uniform sound field with a single driving frequency can be challenging. Moreover, even devices driven by the same type of piezoelectric transducers exhibited variability in the sound fields, primarily due to the manual gluing process and fabrication tolerance in chip manufacturing. Nevertheless, all six devices tested in this study demonstrated high efficiency, generating strong sound fields at low *P*_in_. These devices are ideal for high‐throughput applications, where strong sound fields can be achieved without significant temperature rise. We then performed the particle focusing experiments using 5‐μm‐diameter polystyrene particles with one PZT device and one BNT‐BT‐BNMN device at high flow rates *Q* under *P*_in_ = 1 W, as shown in **Figure** **4**. The particle‐focusing band clearly saturated at this *P*_in_ when *Q* ⩽ 4 mL min^−1^ for both devices, as evidenced by the very small (<3%) normalized full width at half maximum (FWHM) $\tilde{B}$ (calculated as $\tilde{B}$ = FWHM/*W*_mid_, where *W*_mid_ = 363 μm is the channel width at mid‐height). When *Q* ⩾ 6 mL min^−1^, the $\tilde{B}$ value for the PZT device started to increase, while perfect focusing (with $\tilde{B}\leq 5\%$ and 100% recovery from the center outlet) was still achieved by the BNT‐BT‐BNMN device even at *Q* = 10 mL min^−1^. This corresponds to a mean flow velocity of 3.62 m s^−1^ along the channel length (*x*) direction and an averaged fluid retention time of 12.4 ms in the entire channel. This performance surpasses the best particle focusing results reported in previous works on acoustofluidic devices. The clear difference at high *Q* indicates that the induced *E*_ac_, thus the performance, of the BNT‐BT‐BNMN device is superior to that of the PZT device at this power level. Furthermore, the temperature of the PZT transducer saturated at 45 °C under *P*_in_ = 1 W and *Q* = 10 mL min^−1^, while the temperature of the BNT‐BT‐BNMN transducer saturated at 37 °C under the same conditions. In both cases, the channel temperature measured on top of the glass lid stabilized at around 32 °C. These results align with earlier findings that Mn‐doped BNT‐based piezoelectric materials outperform hard PZT at intermediate and high power levels. This includes suppressed nonlinearity in general, the higher mechanical *Q*‐factor *Q*_m_, and less heat dissipation.^[^ ^34^, ^35^, ^36^, ^37^, ^60^ ^]^ A similar trend was not observed in ultrasonic devices using BNT‐based materials without Mn doping,^[^ ^25^, ^31^, ^32^ ^]^ LiNbO_3_,^[^ ^26^ ^]^ KNN‐based materials,^[^ ^28^, ^29^, ^30^ ^]^ or other types of lead‐free piezoelectric materials.^[^ ^27^ ^]^

**Figure 5:**
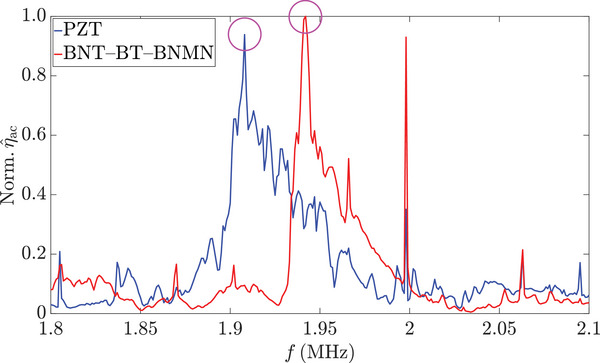
The normalized acoustic performance number ${\hat{\eta }}_{\mathrm{ac}}$, which indicates the device efficiency, was obtained from numerical simulations in the frequency range of 1.8 to 2.1 MHz. The frequency at which ${\hat{\eta }}_{\mathrm{ac}}$ is highest is marked by a magenta circle for each device. A strong lateral mode in the channel length (*x*) direction is excited at *f*=1.998 MHz in both devices, which leads to a peak in the respective ${\hat{\eta }}_{\mathrm{ac}}$ spectrum.

We evaluate the device efficiency in numerical simulations using a dimensionless acoustic performance number, ${\hat{\eta }}_{\mathrm{ac}}=\omega E_{\mathrm{ac}}V/\left(2\pi P_{\mathrm{in}}\right)$, where *ω* and *V* represent the angular frequency and the channel volume where the transducer is attached, respectively. This number was first introduced in an early work of acoustofluidics research,^[^ ^61^ ^]^ but had largely been overlooked until the recent use of a similar number to assess device efficiency.^[^ ^23^, ^62^ ^]^ To maintain consistency with previous work, we adopt the same definition and terminology as in ref. [61]. The definition of ${\hat{\eta }}_{\mathrm{ac}}$ is similar to that of the *Q*‐factor, differing only by a factor of 2π, as it represents the ratio of stored energy to dissipated energy per cycle. However, it is important to note that ${\hat{\eta }}_{\mathrm{ac}}$ is not exactly the same as *Q*‐factor. To calculate the *Q*‐factor in the fluid *Q*_f_, the denominator should correspond to the dissipated power in the fluid, whereas *P*_in_ represents the total dissipated power in both the solid and fluid domains. This distinction highlights an interesting characteristic of acoustofluidic devices, i.e. only the stored energy in the fluid is useful, while the stored energy in the solid is considered as a loss, even though the acoustic field in the fluid is excited by the vibration of the solid. Thus, in an ideal scenario, an efficient BAW device should utilize a mode that generates strong acoustic energy in the fluid with minimal vibration in the solid. When comparing ${\hat{\eta }}_{\mathrm{ac}}$ across the same frequency range (see **Figure** **5**), we find that the highest ${\hat{\eta }}_{\mathrm{ac}}$ values of the PZT and BNT‐BT‐BNMN devices are very similar, indicating comparable device efficiency. This trend from the simulation aligns well with the experimental results at low power, where *E*_ac_ was measured under the same *P*_in_.

It is surprising that the BAW devices driven by PZT and BNT‐BT‐BNMN exhibit similar device performance at low power, given that the clearly inferior piezoelectric properties of BNT‐BT‐BNMN. Two possible reasons contributing to this are examined here. The acoustic impedance of Pz26 and HC‐70BN are *Z*_PZT_ = 2.77 × 10^7^ and *Z*_BNT_ = 2.48 × 10^7^ Pa · s m^−3^ (the longitudinal speed of sound was estimated from the frequency constant of the 33‐mode *N*_33_), respectively, while the acoustic impedance for Pyrex glass is *Z*_Pyrex_ = 1.26 × 10^7^ Pa · s m^−3^. Consequently, the energy transmission coefficient at the interface between PZT and Pyrex is 0.8602, while for BNT‐BT‐BNMN and Pyrex, it is 0.895, calculated using the formula *T* = 4*Z*_1_ *Z*_2_/(*Z*_1_ + *Z*_2_)^2^. Therefore, more acoustic energy can be transmitted from the BNT‐BT‐BNMN transducer to the chip compared to the PZT‐driven device. The lower acoustic impedance of BNT‐BT‐BNMN is primarily due to its low density, as lead, which has a high density of 11 348 kg m^−3^, is absent in BNT‐BT‐BNMN.

**Figure 6:**
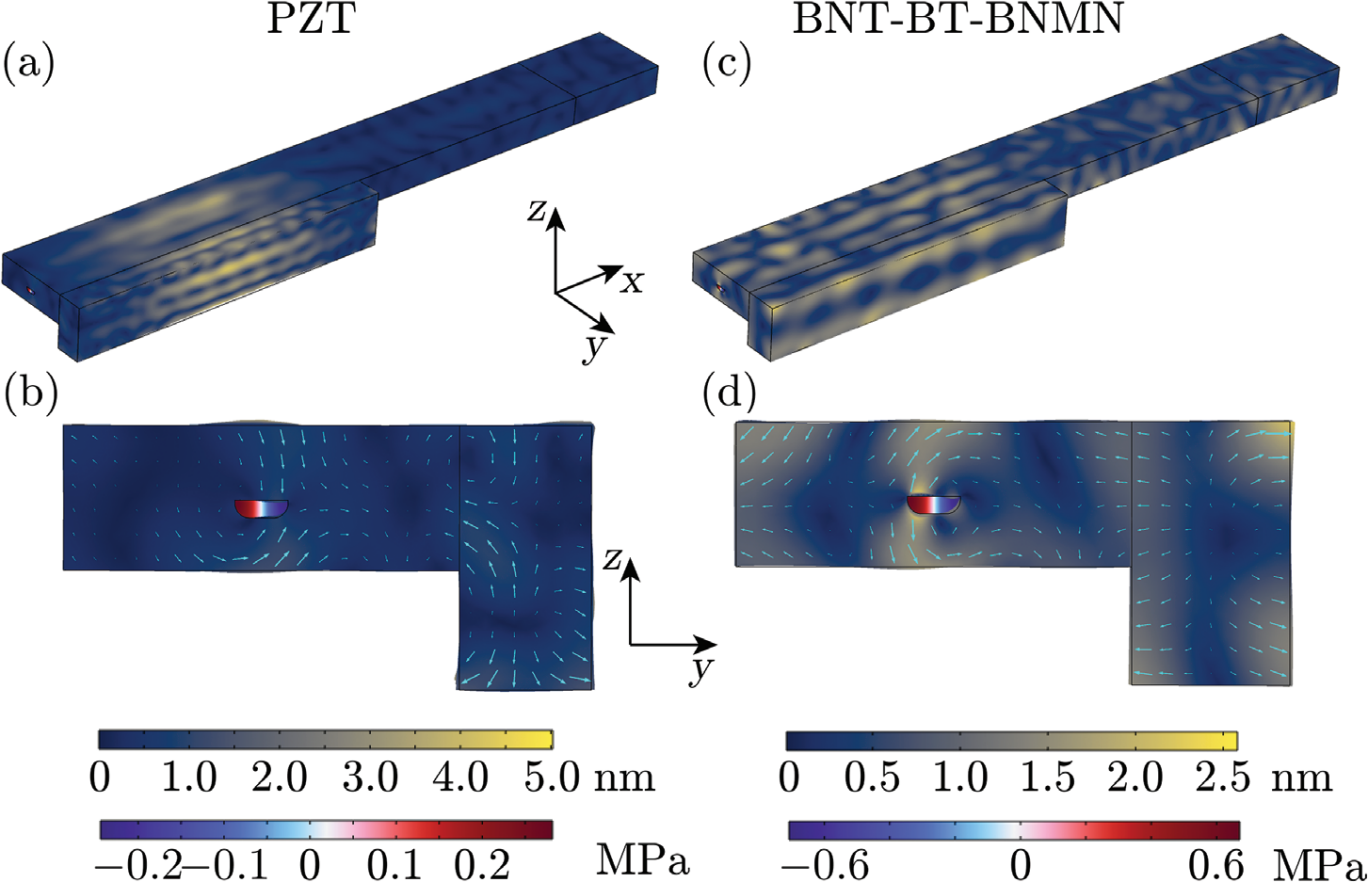
Results of 3D numerical simulations for devices driven by PZT and BNT‐BT‐BNMN, evaluated at frequencies *f*_PZT_ = 1.908 MHz and *f*_BNT_ = 1.942 MHz, under an electric potential of 2.5 V across the transducers. a) Color plot of the displacement field in the solid [magnitude from 0 (dark blue) to 5.0 nm (yellow)] and the acoustic pressure field in the fluid [from −0.25 MPa (blue) to 0.25 MPa (red)] for the PZT device. b) Cross‐sectional view (*yz*‐plane) of the PZT device, with the displacement amplitude and direction indicated by cyan arrows. c) Color plot of the displacement field in the solid [magnitude from 0 (dark blue) to 2.5 nm (yellow)] and the acoustic pressure field in the fluid [from −0.6 MPa (blue) to 0.6 MPa (red)] for the BNT‐BT‐BNMN device. d) Corresponding cross‐sectional view of the BNT‐BT‐BNMN device, with the displacement amplitude and direction indicated by cyan arrows.

**Figure 7:**
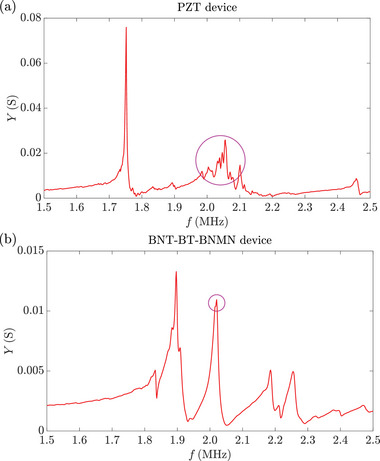
Measured admittance (*Y*) spectra of a) the PZT and b) the BNT‐BT‐BNMN devices. In the admittance spectrum of the PZT device, multiple resonance peaks appeared close to each other at around 2 MHz, resulting from the overlap of the longitudinal and transverse modes induced in the PZT transducer. In contrast, the admittance spectrum of the BNT‐BT‐BNMN device showed a distinct peak due to the weak transverse mode that the BNT‐BT‐BNMN transducer excited.

Despite the higher energy transmission coefficient between BNT‐BT‐BNMN and Pyrex, the 4% difference in *T* is not enough to compensate for the disparity in the piezoelectric properties between PZT and BNT‐BT‐BNMN. Another potential reason lies in a unique characteristic of BNT‐BT‐BNMN, i.e. the transverse mode it excites is very weak. In most BAW devices, the longitudinal waves generated by the piezoelectric transducers are primarily used to excite the acoustic field in the channel. Similarly, in most ultrasonic devices, the longitudinal mode is the dominating mode, as the piezoelectric charge constant and the electromechanical coupling coefficient of the longitudinal mode *d*_33_ and *k*_33_ are considerably higher than those of the transverse mode (*d*_31_ and *k*_31_) in piezoelectric materials, which is true for both PZT and BNT‐BT‐BNMN. As a result, BAW devices are typically designed to match the resonance frequency of the transducer's thickness mode with the resonance frequency in the channel. However, in practice, transverse waves are also excited and mixed with the longitudinal waves due to the large dimensions of the transducer in the length (*x*) and width (*y*) directions, which allow transverse waves to propagate with multiple wavelength. This phenomenon is evident in both the vibration mode of the PZT transducer obtained from simulation and the measured admittance spectrum, as shown in **Figures** **6**a,b and **7**a. The vibration mode of the PZT transducer clearly shows transverse waves, indicated by the multiple ripples along the *x*‐ and *y*‐directions (see Video S1, Supporting Information). As a result, the admittance spectrum does not exhibit a clean peak around 2 MHz (see Figure 7a), as different modes overlap and mix. When two modes overlap, both consume input electric energy, leading to lower vibration amplitudes in the transducer than would occur if only the longitudinal mode was excited under the same *P*_in_. In contrast, BNT‐BT‐BNMN has an extremely low *k*_31_ (0.09), meaning that very little input electric energy is converted into the mechanical vibration in the transverse mode. This is evidenced by the much smoother vibration mode in the BNT‐BT‐BNMN transducer with weaker ripples, as illustrated in Figure 6c and Video S3 (Supporting Information), as well as the clean peak in the admittance spectrum (see Figure 7b). Additionally, the vibration component of the thickness mode in the BNT‐BT‐BNMN transducer is much stronger in the *yz*‐plane, as shown in Figure 6d. If a pure longitudinal mode was excited, the performance of the PZT transducer would outperform that of the BNT‐BT‐BNMN transducer at low power. This is demonstrated by simulations of piezoelectric cylinders made from both materials at their fundamental longitudinal resonance frequencies, as discussed in Section S3 (Supporting Information). Although different vibration modes were excited in PZT and BNT‐BT‐BNMN transducers, which led to distinct vibration modes in the chip, the vibration mode of the channel walls was quite similar in both cases (see Videos S2 and S4, Supporting Information). This similarity arises from the acoustic impedance mismatch between the solid and fluid, which isolates the resonance mode in the fluid from that in the solid. Consequently, different solid vibration modes lead to similar resonance modes in the fluid, as long as the driving frequency is close to the resonance frequency in the fluid for a particular resonance mode. To summarize, we believe the weak transverse mode excited by BNT‐BT‐BNMN is the primary reason why BNT‐BT‐BNMN devices perform similarly to PZT devices at low power, despite the inferior piezoelectric properties of BNT‐BT‐BNMN. The unique properties of BNT‐based piezoelectric materials have not been explored in ultrasonic devices in previous studies.

## 5 Conclusion

In this work, we experimentally and numerically compared the performance of BAW acoustofluidic devices driven by PZT and BNT‐BT‐BNMN transducers. By comparing the acoustic energy density measured using μPIV under the same input power and evaluating the device efficiency through numerical simulations, we found that the devices driven by PZT and BNT‐BT‐BNMN exhibit comparable performance at low power levels, despite the inferior piezoelectric properties of BNT‐BT‐BNMN. The high performance of the BNT‐BT‐BNMN devices at low power is attributed to two factors. First, the higher energy transmission coefficient between BNT‐BT‐BNMN and Pyrex glass compared to that of PZT and Pyrex interface enables more efficient transmission of wave energy from the transducer to the device. Second, the weak transverse waves excited in the BNT‐BT‐BNMN transducer, due to its low electromechanical coupling factor of the transverse mode *k*_31_, ensure that most of the input electric energy is converted into longitudinal waves, which is more efficient for driving the device. The BNT‐BT‐BNMN device demonstrated perfect focusing of 5‐μm‐diameter polystyrene particles at flow rates up to 10 mL min^−1^ under input power of 1 W, outperforming the PZT device under the same conditions. This superior performance under intermediate power levels is due to the better piezoelectric properties of Mn‐doped BNT‐based materials under intermediate and high power levels, which aligns with previous findings on piezoelectric materials. The results of this work demonstrate that acoustofluidic devices can simultaneously offer high efficiency, capability of performing high‐throughput applications, good biocompatibility, and environmental friendliness. Given that most BAW acoustofluidic devices, including those studied here, utilize the thickness mode of the piezoelectric transducers (such as planar resonant devices,^[^ ^33^, ^63^, ^64^ ^]^ acoustic trapping devices,^[^ ^13^, ^65^ ^]^ monolithic acoustic holograms,^[^ ^7^, ^66^ ^]^ and devices using transducer arrays),^[^ ^67^, ^68^ ^]^ the findings from this work should be broadly applicable when replacing PZT with BNT‐BT‐BNMN in various BAW devices. Furthermore, these positive results with BNT‐BT‐BNMN transducers may influence the acoustofluidics industry, particularly the commercialization of lead‐free BAW devices progresses to comply with the legislations. This study also provides compelling evidence that PZT can be replaced by lead‐free piezoelectric materials in ultrasonic actuation devices without compromising performance across different power ranges, the impact of which extends beyond the acoustofluidics field. We hope that this work will help guide the future development of high‐performance, environmentally‐friendly acoustofluidic devices for a variety of applications.

## Conflict of Interest

The authors declare no conflict of interest.

## Supporting information

Supporting Information

Supplemental Video 1

Supplemental Video 2

Supplemental Video 3

Supplemental Video 4

## Data Availability

The data that support the findings of this study are available from the corresponding author upon reasonable request.
