# Supplementary material for: High‐Performance and Environmentally‐Friendly Bulk‐Wave‐Acoustofluidic Devices Driven by Lead‐Free Piezoelectric Materials
Source: Small. 2024 Nov 24;21(10):2407453. doi: 10.1002/smll.202407453 (PMC11899509; doi:10.1002/smll.202407453)
Supplement: Supplementary file 1 — Supporting Information [file SMLL-21-2407453-s005.pdf]

NANO · MICRO
small

Supporting Information

for *Small*, DOI 10.1002/smll.202407453

High-Performance and Environmentally-Friendly Bulk-Wave-Acoustofluidic Devices Driven by Lead-Free Piezoelectric Materials

*Wei Qiu**

Supporting Information

High-Performance and Environmentally-Friendly Bulk-Wave-Acoustofluidic Devices Driven by Lead-Free Piezoelectric Materials

*Wei Qiu**

S1. Photograph of the experimental setup

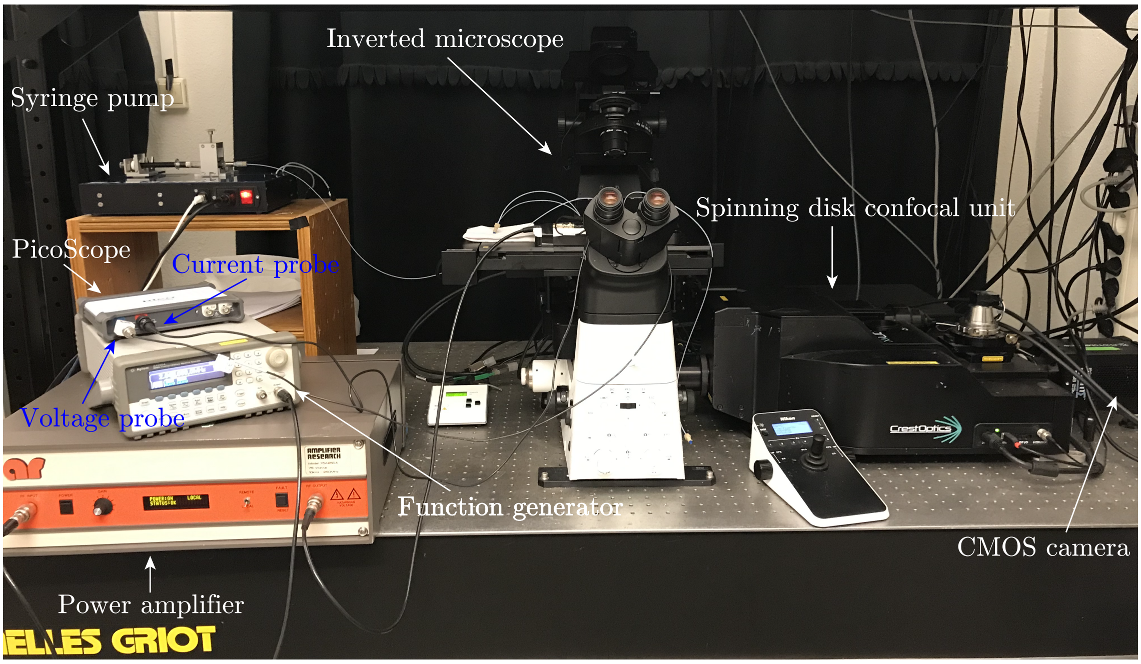

Figure S1. Photograph of the experimental setup. The acoustofluidic device was mounted on an inverted microscope equipped with a spinning-disk confocal unit and a CMOS camera. The device was connected to a syringe via the Teflon tubing, and a syringe pump was used to infuse the particle solutions. For low-power experiments, the device was driven directly by a function generator, while the driving signal was amplified by a power amplifier for intermediate-power experiments. The voltage and current supplied to the transducer were measured using voltage and current probes, with the data monitored via a PicoScope, allowing for the calculation of input power. The light source (a laser diode) is not shown in this photograph.

S2. Mesh convergence analysis

To assess the appropriateness of the mesh element size in our 3D numerical model, we performed a mesh convergence analysis following the approach outlined by Muller et al.[1] A relative convergence parameter $C(g)$ was defined as

$$C(g) = \sqrt{\frac{\int (g - g_{\mathrm{ref}})^2 dV}{\int (g_{\mathrm{ref}})^2 dV}} \quad \text{(S1)}$$

for a solution g with respect to a reference solution g_{ref} calculated with the smallest mesh element size. The full-device model consists of several domains, including water, glass, and transducer, each requiring different mesh element sizes. A common method to evaluate mesh element sizes across these domains is to define $h_{\mathrm{domain}} = \lambda_{\mathrm{domain}}/N$, where h_{domain} is the maximum mesh element size in a given domain, $\lambda_{\mathrm{domain}}$ is the wavelength in the same domain, and N is the number of mesh elements per wavelength.[2, 3] By varying N, mesh element sizes across the different domains can be evaluated simultaneously. However, a limitation of this approach is that the mesh may converge for different values of N in different domains, potentially resulting in overly fine meshes in some domains.

In this study, we conducted the mesh convergence analysis differently. We first evaluated mesh convergence in the transducer domain (only the analysis for the PZT device is shown here, as the procedure is identical for the BNT-BT-BNMN device). The maximum mesh element sizes

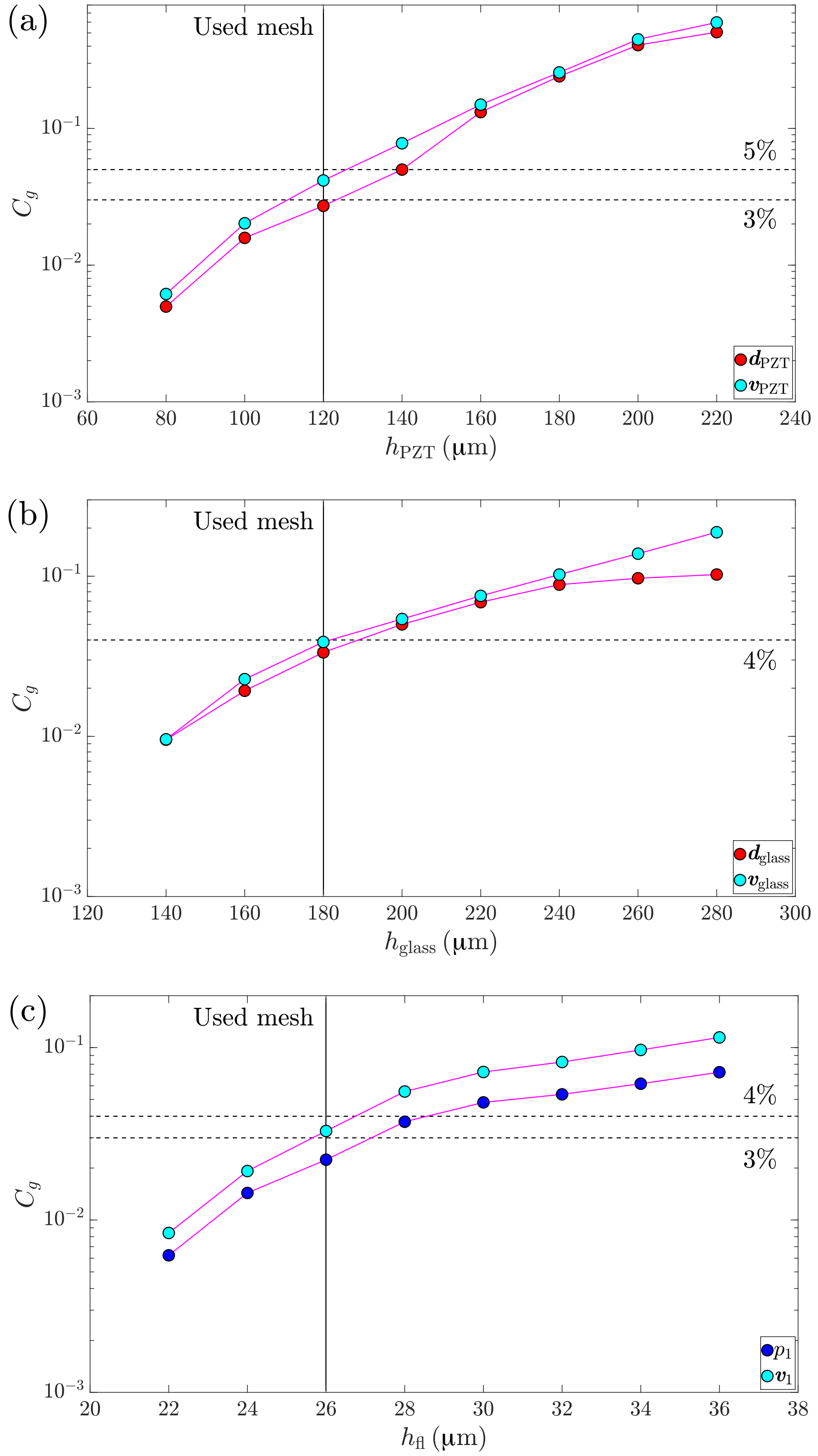

Figure S2. Semi-logarithmic plot of the relative convergence parameter $C(g)$ in (a) the transducer (PZT) domain, (b) the glass domain, and (c) the fluid domain. The displacement and velocity fields (***d*** and ***v***) were evaluated in the transducer and glass domains, while the first-order pressure and velocity fields (p_1 and $\boldsymbol{v}_1$) were assessed in the fluid domain. In plot (a), the maximum mesh element sizes in glass and fluid domains were fixed to h_{glass} = 180 µm and h_{fl} = 26 µm, while the smallest maximum mesh element size in PZT domain, $h_{\text{PZT}}^{\text{min}}$, was 60 µm. In plot (b), h_{PZT} and h_{fl} were fixed at 120 µm and 26 µm, while $h_{\text{glass}}^{\text{min}}$ = 120 µm. In plot (c), h_{PZT} and h_{glass} were fixed to 120 µm and 180 µm, while $h_{\text{fl}}^{\text{min}}$ = 20 µm.

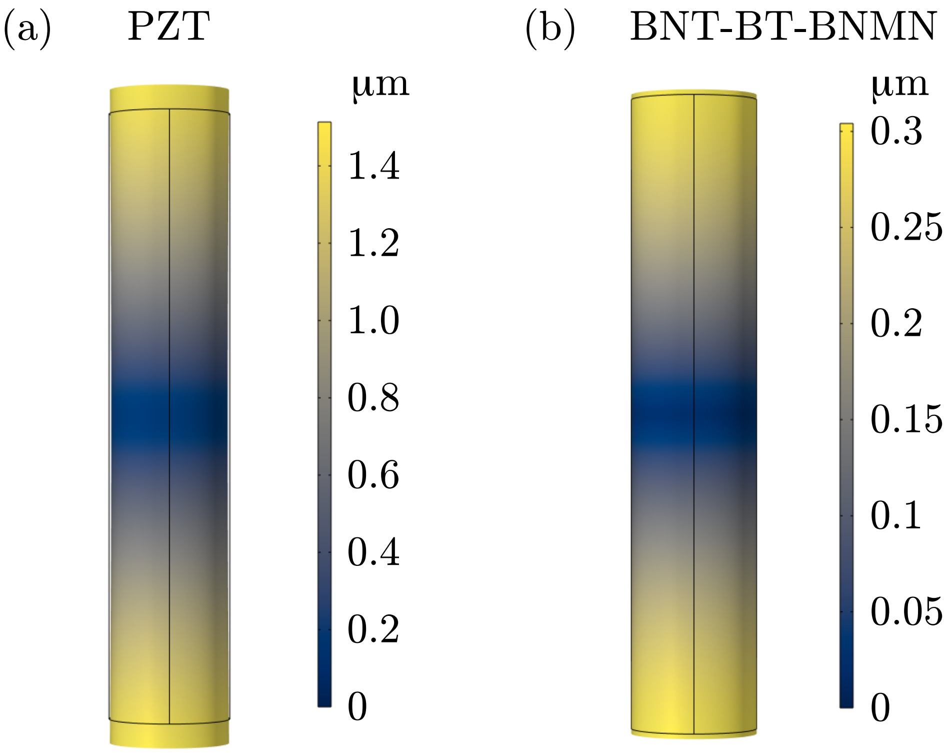

Figure S3. Simulated longitudinal vibration mode at the fundamental resonance frequency for (a) a PZT cylinder and (b) a BNT-BT-BNMN cylinder. The input power to both transducers was set to 50 mW.

in the glass and fluid domains were fixed at h_{glass} = 180 µm and h_{fl} = 26 µm, respectively, as shown in Figure S2(a). We then assessed the displacement and velocity fields in the PZT transducer, devoted $\boldsymbol{d}_{PZT}$ and $\boldsymbol{v}_{PZT}$. The optimal mesh element size in PZT domain, h_{PZT} = 120 µm, was found, yielding $C(g)$ of less than 5% for both $\boldsymbol{d}_{PZT}$ and $\boldsymbol{v}_{PZT}$. Next, we evaluated h_{glass} by fixing h_{PZT} = 120 µm and h_{fl} = 26 µm [see Figure S2(b)]. At h_{glass} = 180 µm, $C(g)$ was below 4% for both the displacement and velocity fields in the glass domain, $\boldsymbol{d}_{glass}$ and $\boldsymbol{v}_{glass}$, indicating that this mesh size is appropriate for the glass domain. If the mesh does not converge around h_{glass} = 180 µm, it suggests that the initial guess is incorrect, and the mesh convergence analysis in the transducer domain should be repeated with a different h_{glass} value. The same procedure was applied when evaluating mesh convergence in the fluid domain.

S3. Simulation of piezoelectric cylinders

To compare the performance of transducers when the pure longitudinal mode is excited, we conducted 3D numerical simulations on piezoelectric cylinders made of PZT and BNT-BT-BNMN, as shown in Figure S3. The cylinder had a diameter of 10 mm and a height of 50 mm, which allows for the excitation of a pure longitudinal mode without interference from the transverse mode. The polarization direction was aligned with the height of the cylinder. Zero stress was applied to the outer boundaries facing the air, and an alternating electric potential was applied through the electrodes on the top and bottom surfaces of the transducer. The electric potential was adjusted so that the input power to both the PZT and BNT-BT-BNMN transducers was 50 mW. At the fundamental resonance frequency of the longitudinal mode, the induced vibration amplitude of the PZT cylinder was consierably higher than that of the BNT-BT-BNMN cylinder. This suggests that, when designed to operate in a pure longitudinal mode, the PZT transducer outperforms the BNT-BT-BNMN transducer due to PZT's superior piezoelectric properties at low power levels. However, it is important to note that this

conclusion applies only to low-power conditions. Under intermediate and high power levels, BNT-BT-BNMN performs better than PZT, even when the pure longitudinal mode is excited. [4]

Video S1. Time-harmonic animation of Figure 6(a) that shows the displacement field in the solid [magnitude from 0 (dark blue) to 5.0 nm (yellow)] and the acoustic pressure field in the fluid [from -0.25 MPa (blue) to 0.25 MPa (red)] in 3D for the PZT device.

Video S2. Time-harmonic animation of Figure 6(b) that shows the cross-sectional view of the PZT device with the displacement amplitude and direction indicated by cyan arrows.

Video S3. Time-harmonic animation of Figure 6(c) that shows the displacement field in the solid [magnitude from 0 (dark blue) to 2.5 nm (yellow)] and the acoustic pressure field in the fluid [from -0.6 MPa (blue) to 0.6 MPa (red)] in 3D for the BNT-BT-BNMN device.

Video S4. Time-harmonic animation of Figure 6(d) that shows the cross-sectional view of the BNT-BT-BNMN device with the displacement amplitude and direction indicated by cyan arrows.

References

[1] Peter B. Muller, Rune Barnkob, Mads J. H. Jensen, and Henrik Bruus, A numerical study of microparticle acoustophoresis driven by acoustic radiation forces and streaming-induced drag forces, Lab Chip **12**, 4617 (2012).
[2] Mikkel W. H. Ley and Henrik Bruus, Three-dimensional numerical modeling of acoustic trapping in glass capillaries, Phys. Rev. Appl. **8**, 024020 (2017).
[3] Wei Qiu, Jonas H. Joergensen, Enrico Corato, Henrik Bruus, and Per Augustsson, Fast microscale acoustic streaming driven by a temperature-gradient-induced nondissipative acoustic body force, Phys. Rev. Lett. **127**, 064501 (2021).
[4] Tonshaku Tou, Yuki Hamaguti, Yuichi Maida, Haruo Yamamori, Kazutoshi Takahashi, and Yoshimitsu Terashima, Properties of $(Bi_{0.5}Na_{0.5})TiO_3$-$BaTiO_3$-$(Bi_{0.5}Na_{0.5})(Mn_{1/3}Nb_{2/3})O_3$ lead-free piezoelectric ceramics and its application to ultrasonic cleaner, Jpn. J. Appl. Phys. **48**, 07GM03 (2009).
